# Applied machine learning to identify differential risk groups underlying externalizing and internalizing problem behaviors trajectories: A case study using a cohort of Asian American children

**DOI:** 10.1371/journal.pone.0282235

**Published:** 2023-03-03

**Authors:** Samrachana Adhikari, Shiying You, Alan Chen, Sabrina Cheng, Keng-Yen Huang

**Affiliations:** Department of Population Health, New York University Grossman School of Medicine, New York, NY, United States of America; Yale University, UNITED STATES

## Abstract

**Background:**

Internalizing and externalizing problems account for over 75% of the mental health burden in children and adolescents in the US, with higher burden among minority children. While complex interactions of multilevel factors are associated with these outcomes and may enable early identification of children in higher risk, prior research has been limited by data and application of traditional analysis methods. In this case example focused on Asian American children, we address the gap by applying data-driven statistical and machine learning methods to study clusters of mental health trajectories among children, investigate optimal predictions of children at high-risk cluster, and identify key early predictors.

**Methods:**

Data from the US Early Childhood Longitudinal Study 2010–2011 were used. Multilevel information provided by children, families, teachers, schools, and care-providers were considered as predictors. Unsupervised machine learning algorithm was applied to identify groups of internalizing and externalizing problems trajectories. For prediction of high-risk group, ensemble algorithm, Superlearner, was implemented by combining several supervised machine learning algorithms. Performance of Superlearner and candidate algorithms, including logistic regression, was assessed using discrimination and calibration metrics via crossvalidation. Variable importance measures along with partial dependence plots were utilized to rank and visualize key predictors.

**Findings:**

We found two clusters suggesting high- and low-risk groups for both externalizing and internalizing problems trajectories. While Superlearner had overall best discrimination performance, logistic regression had comparable performance for externalizing problems but worse for internalizing problems. Predictions from logistic regression were not well calibrated compared to those from Superlearner, however they were still better than few candidate algorithms. Important predictors identified were combination of test scores, child factors, teacher rated scores, and contextual factors, which showed non-linear associations with predicted probabilities.

**Conclusions:**

We demonstrated the application of data-driven analytical approach to predict mental health outcomes among Asian American children. Findings from the cluster analysis can inform critical age for early intervention, while prediction analysis has potential to inform intervention programing prioritization decisions. However, to better understand external validity, replicability, and value of machine learning in broader mental health research, more studies applying similar analytical approach is needed.

## Introduction

The burden of mental, neurological, and substance disorders account for 10–18% of the Global Burden of Disease [1, 2], and half of the cases develop by age of 14 [3, 4]. Internalizing problems (anxiety, depression) and externalizing problems (aggressive behavior, conduct difficulties) together account for over 75% of the total mental health burden in children and adolescents [4, 5]. Mental health burden among children and adolescents from racial and ethnic minorities in the US is even higher (31–45%) [3, 6, 7]. However, knowledge related to the patterns of developmental trajectories during the critical childhood periods and factors that contribute to the disorders in different ethnic minorities remains limited. Although progress has been made in using a more comprehensive Health Disparities Framework to better inform mental health disparities research [8], analytical tools used in such research are still largely traditional, limiting further progress.

Most health disparities research has relied on data with small sample size using conventional regression approach that relies on strong modeling assumptions for analysis. However, such analytical approach can be limited when incorporating multidomain and multilevel risk factors simultaneously, leading to suboptimal models with less reliable results and limiting the generalizability and predictive ability of the findings, making them less actionable. Thus, to advance actionable mental health disparities research, application of modern data-driven statistical approaches and predictive models utilizing rich multidomain contextual and longitudinal data must be explored. Applications of data-driven analytical approaches can help us utilize predictive tools that are often more suitable for large datasets with many predictors, and may also inform personalized health intervention decisions and support better understanding of mental health developmental processes.

This paper addressed the gaps in mental health disparities and developmental psychopathology research by applying modern statistical and machine learning methods to study patterns of mental health trajectories from early childhood to early teen periods (kindergarten to Grade 5) and investigate key early predictors (i.e., individual, parental and contextual factors at kindergarten) of poor and optimal trajectories. We explored the feasibility and potential benefits, if any, of using big administrative data from a national longitudinal survey along with data-driven machine learning (unsupervised and supervised) algorithms in child and adolescent mental health research. Unsupervised machine learning algorithms can help identify underlying sub-groups in the trajectories of internalizing and externalizing problem behaviors, whereas supervised machine learning algorithms can optimize predictions by utilizing high dimensional predictors with minimal prior assumptions on the models and produce highly accurate and well-calibrated predictions.

Considering different ethnic populations’ unique living experiences and contexts, which may associate with different mechanisms toward disparities, this study focused on Asian American children and adolescents as a case example to demonstrate the application of machine learning in mental health disparities research. We focused on Asian Americans for three reasons. First, prior studies have documented that while Asian American children and adolescents were at greater risk for internalizing problems, they had similar risks for externalizing problems, relative to non-Asian US children and Non-US Asian children [6, 9]. Approximately one third of Asian American children were at elevated risk for anxiety, somatization, and depressive problems, relative to 12–15% reported for the general population in the literature [6, 9]. Second, a combination of cultural, individual, family, and school factors, which explained 17–39% of the variance in mental health problem development in Asian American children and adolescents, have been studied in the literature [10–13], including our prior work [6, 14, 15]. This study builds on prior work and considers multi-level factors and longitudinal mental health data simultaneously to document the added values in knowledge gain using machine learning. Third, since Asian-Americans are one of the fastest-growing ethnic minorities in the US, with a population of around 20 million [16], understanding the health status and service needs of this population is imperative to advance population health.

In this paper, we first investigated heterogeneity on the longitudinal trajectory of mental health problem behaviors among Asian American children and assess clusters of children in the high-risk category. Second, applying multi-dimensional risk factors data (individual, parental, and contextual factors) at kindergarten and ensemble of supervised machine learning algorithms, we evaluated prediction algorithms for identification of Asian American children at high-risk. We hypothesized that the machine learning algorithms will have better predictive performance than the traditional parametric logistic regression.

## Materials and methods

### Data source

This study leveraged a rich publicly available national database from the “Early Childhood Longitudinal Study—Kindergarten Class” (ECLS-K) 2011 cohort [17]. The ECLS-K study, which was sponsored by the US Department of Education’s National Center for Education Statistics, followed a cohort of children from kindergarten through their elementary school years, using a multistage probability sampling design, to select a nationally representative sample of children attending kindergarten in the academic year 2010–2011. Approximately 18,200 children (and parents) throughout the country were recruited. Three primary methods of data collection, direct child assessment, parent interview, and teacher and school administrator questionnaires, were utilized. Current analysis was based on the public-use dataset, utilizing ECLS-K 2011 sub-cohort of 1,660 children with self-reported race of Asian or Native Hawaiian/Pacific Islander. Further details on data collection methodology are published elsewhere [17].

### Measures

#### Externalizing and internalizing problem behaviors subscales

The Social Rating Scale (SRS) adapted from the Social Skills Rating System (SSRS [18]) was used to assess the mental health status of children in a classroom, starting in kindergarten. The SRS is based on teacher ratings on a four-point scale, which includes subscales on (a) Externalizing Problem Behaviors; (b) Internalizing Problem Behaviors; (c) Self-Control; and (d) Interpersonal Skills. Teachers used a frequency scale (1–4) to rate how often a child displays a particular social skill or behavior (i.e., 1 = a student never exhibits this behavior; 4 = a student exhibits this behavior most of the time). Our outcome measures included two subscales, externalizing and internalizing problem behaviors, from the SRS collected over six years.

The externalizing problem behaviors measure acting out behaviors, such as arguing, fighting, showing anger, acting impulsively, and disturbing the classroom’s ongoing activities. Whereas, the internalizing problem behaviors measure whether a child appears anxious, lonely, sad, or has low self-esteem. Scores for the externalizing and internalizing problem behaviors sub-scales were reported in fall and spring periods of kindergarten through the second grade (2010–2013), and in the spring periods of third through fifth grades (2014–2016). Due to low response rates in the fall periods, only spring period measurements were considered in our analysis. When possible, fall scores were carried forward to impute missing spring scores in the same grade for both problem behaviors.

#### Race/ethnicity

Race/ethnicity data were collected from parents’ self-report. While the information on subgroups within Asian American race was not available in the public-use dataset considered for this paper, restricted-use data included such information. The composition of race/ethnicity, which has been published elsewhere [19], suggests that the Asian race in ECLSK-2011 included seven subgroups within the Asian American community: Indian, Chinese, Hmong, Japanese, Korean, Vietnamese, and other Asian. Thus, the cohort of Asian Americans for this analysis consisted of diverse representative groups, also including Pacific Islanders and multiethnic Asians, irrespective of ethnicity [20].

#### Baseline predictors

Multidomain/Multilevel contextual variables assessed at kindergartens were used as predictors. We selected representative variables, instead of an exhaustive list, based on the social determinants framework for the purpose of exploration and feasibility demonstration. Children, their families, teachers, schools and care providers provided information on children’s cognitive, social, emotional, and physical development characteristics, as well as culture, immigration, home environment, parenting, home educational activities, school environment and quality, and neighborhood characteristics.

Child level predictors included measures on child learning, health, physical and socioemotional wellbeing, disability status, and social-process skills from direct child assessment, and parent and teacher report data. The kindergarten direct child assessment measured reading, mathematics, and science knowledge and skills, and executive function. Teachers reported on approaches to learning scale, providing information on how often their students exhibited a selected set of learning behaviors. The kindergarten questionnaires also asked teachers to indicate how often their children exhibited certain social skills and behaviors related to inhibitory control and attentional focusing. The parents-reported social scales consisted of four subscales, self-control, social interaction, sad/lonely, and impulsive/overactive behaviors. Parents also reported on approaches to learning scale. The parent interview reported on family structure, family literacy practices, parental involvement in school, care arrangements, household composition, family income, parent education level, culture/immigration, and other demographic indicators. Neighborhood level measures included measures of community support and community violence.

A complete list of predictors and their descriptions are presented in Supplement 1. Measures with less than 30% missingness were included in the predictive analysis, resulting in a total of 24 predictors. Continuous predictors were standardized to have mean 0 and variance of 1. Mean imputation was considered for missing continuous variables. An explicit class of missing was introduced for each categorical variable to maintain sample size in the predictive analysis.

### Statistical analysis

#### Longitudinal trajectory analysis

To investigate heterogeneity in the trajectory patterns of mental health problems, we applied the latent class mixed effects models (LCMMs) [21], a type of unsupervised algorithm. LCMM, an extension of the linear mixed model or latent class analysis, is a flexible probabilistic approach to uncover underlying clusters of trajectories using longitudinal observations, and allows modeling of various types of data distribution and complex trajectory shapes. Measurements from six time points were used to identify clusters of outcome trajectories. Time was considered as a continuous variable and specified as a fixed effect. Random effects at the child level allowed for within child clustering over time. Multiple LCMMs with several link functions (linear, beta, and I-splines), as well as a linear model with a quadratic time trend were fitted to identify the best fitting model with an optimal number of clusters. To determine the optimal number of clusters and optimal link function, Bayesian information criterion (BIC) was used. Additionally, to avoid models with small class sizes (<5% of total sample), class membership size was also considered while selecting the optimal number of classes. The classes identified from LCMMs were considered as outcome categories in the predictive modeling. Similar to the trajectory analysis, we conducted prediction separately for the internalizing and externalizing problems behaviors.

#### Prediction model development

To predict Asian American children at elevated risk for poor mental health development, we used SuperLearner [22] algorithm, a supervised machine learning approach. Different from the traditional parametric logistic regression model, SuperLearner is a non-parametric prediction method which is constructed as a combination (ensemble) of multiple independent candidate prediction algorithms. By creating an optimal weighted average of predictions from candidate algorithms, with the goal of maximizing area under the curve (AUC), Superlearner performs as well or better compared to the individual algorithms with respect to the loss function (AUC). We considered candidate algorithms that are commonly used for prediction and are suitable for data with a relatively large number of potentially correlated predictors. These candidate algorithms included a simple mean model which predicts the observed proportion of the target outcome class (*mean model*), a traditional logistic regression without penalty (*logistic regression*), a logistic regression with lasso penalty [23] (*lasso regression*), a logistic regression with group lasso penalty [24] (*group lasso regression*), a support vector machine with radial basis kernel [25] (*SVM*), and random forest [26] with nine different parameter settings.

In the mean learner, predicted probability is computed as the percentage of ones in the training data. The traditional logistic regression computes predicted probability of the outcome by fitting a generalized linear model on the training data using a logistic link function. Maximizing a likelihood in the logistic regression is equivalent to minimizing a negative likelihood loss function without any penalty. The lasso and group lasso regressions are extensions of the logistic regression model, such that a penalty is introduced in the loss function. The penalty, which is a function of a user provided hyperparameter, controls the model complexity by shrinking some of the individual coefficients (in lasso regression) or a group of related coefficients (in group lasso regression) to zero. The SVM is a flexible prediction algorithm in which the predictors are mapped into a higher dimensional space using a user defined kernel function to obtain an optimal prediction rule. Finally, the random forest is a tree-based algorithm that produces optimal prediction by aggregating decision trees. Number of trees and number of variables sampled at each split in a tree are the hyperparameters that need to be specified by a user in the random forest.

We selected the hyperparameters for the lasso and group lasso regressions via hyperparameter tuning. The grouping indicator for the group lasso penalty was specified by including the continuous predictors into separate groups and grouping categories from respective categorical predictors together into a group. We specified a radial basis kernel function with default parameter values for the SVM. We considered random forests with combinations of number of trees (ntree) and number of variables sampled at each split (mtry) as different candidate algorithms in the Superlearner. The ntree parameter ranged from 100 to 700 whereas the mtry option was set to 3, 5 and 7. Finally, using optimal fitted weights from the Superlearner, the predicted probability for each individual was computed. Further, as a biproduct of fitting candidate algorithms for creating Superlearner predictions, we were also able to obtain predicted probabilities from each individual algorithm. Thus, to assess prediction performance, we validated and compared predictions from Superlearner and the candidate algorithms.

#### Model validation and assessment of prediction performance

A ten-fold stratified nested crossvalidation (CV) was performed to assess the out-of-sample prediction performance of the algorithms (Superlearner and the candidate algorithms) and for hyper-parameter tuning. The tuning of the hyper-parameters for lasso and group lasso regressions were performed in the inner 10-fold CV. To assess the prediction performance of Superlearner and the candidate algorithms, we considered various metrics. These metrics included accuracy, true positive rate (TPR), true negative rate (TNR), area under the receiver operating characteristic curve (AUC), and area under the precision-recall curve (AUCpr). The probability threshold for prediction of the high-risk class was chosen using Youden’s J statistics [27]. The AUC and AUCpr for Superlearner as well as the candidate algorithms were also compared against baseline values. The baseline value for AUC was 0.5, whereas the baseline value for AUCpr was determined by the ratio of the number of children in the high-risk outcome class over the total number of children, as $\frac{\#\;in\;high\;class}{total\;\#}$. To assess whether the predicted risk scores (out-of-sample predicted probabilities) of problem behaviors correspond to the observed proportions, we evaluated calibrations of the algorithms. Calibration of SuperLearner and the candidate algorithms was assessed visually by plotting distributions of observed proportions and predicted probabilities of the high-risk class within each decile of the predicted probability. An algorithm was considered well-calibrated if the two scores aligned in each decile.

#### Variable importance measures and partial dependence plots

Finally, separate random forest algorithms (with hyperparameters that optimized AUC) were fitted to the entire data to evaluate influential predictors with high variable importance for each outcome. Variable importance was assessed using the mean decrease in Gini index, which was rescaled to 0–100. The mean decrease in Gini index of a variable provides a measure of how often this variable was selected and its overall contribution to the prediction in a random forest. A high value of mean decrease in Gini index indicates high variable importance in the model.

Further to facilitate interpretation, we visualized the dependence of predicted probability on each of the top ten important variables using partial dependence plots [28]. The partial dependence plot shows the marginal effect of a variable on the predicted probability (on logit scale), and can provide insights on the direction of association.

Analyses were conducted in R (version 4.0.2) on MacOS using packages ‘lcmm’, ‘randomForest’, ‘elasticnet’, ‘gglasso’, ‘SuperLearner’, and ‘caret’. Software code developed for the analysis is available as open access in GitHub (https://github.com/shiying88/AAChildMentalHealth).

## Results

### Asian American population characteristics

Children and parents’ demographic characteristics, reading scores at kindergarten, and contextual variables of the analytical cohort are summarized in S1 Table. Of the 1,660 Asian American children, 53% were female, 6.9% had disabilities, and 75% were in kindergarten for the first time while 4% reported repeating kindergarten and 21% did not report this information. Similarly, 48% of the families did not speak English as their primary language at home, 32% of parents had high-school or less than high-school education, 10% of the families made less than 20,000 household annual income, and 4.8% of the families were experiencing food insecurities.

### Patterns of problem behavior trajectories

Children with measurements for less than two time points for either internalizing or externalizing problems scores were dropped from the analysis, resulting in a final analytical sample of 1,279 students. During kindergarten (time 0), the mean internalizing problem score was 1.41 (standard deviation; SD = 0.41) and the mean externalizing problem score was 1.49 (SD = 0.52). By fifth grade, the average internalizing problem score had increased to 1.50 (SD = 0.49) whereas the average externalizing problem score was 1.46 (SD = 0.50).

**Fig 1:**
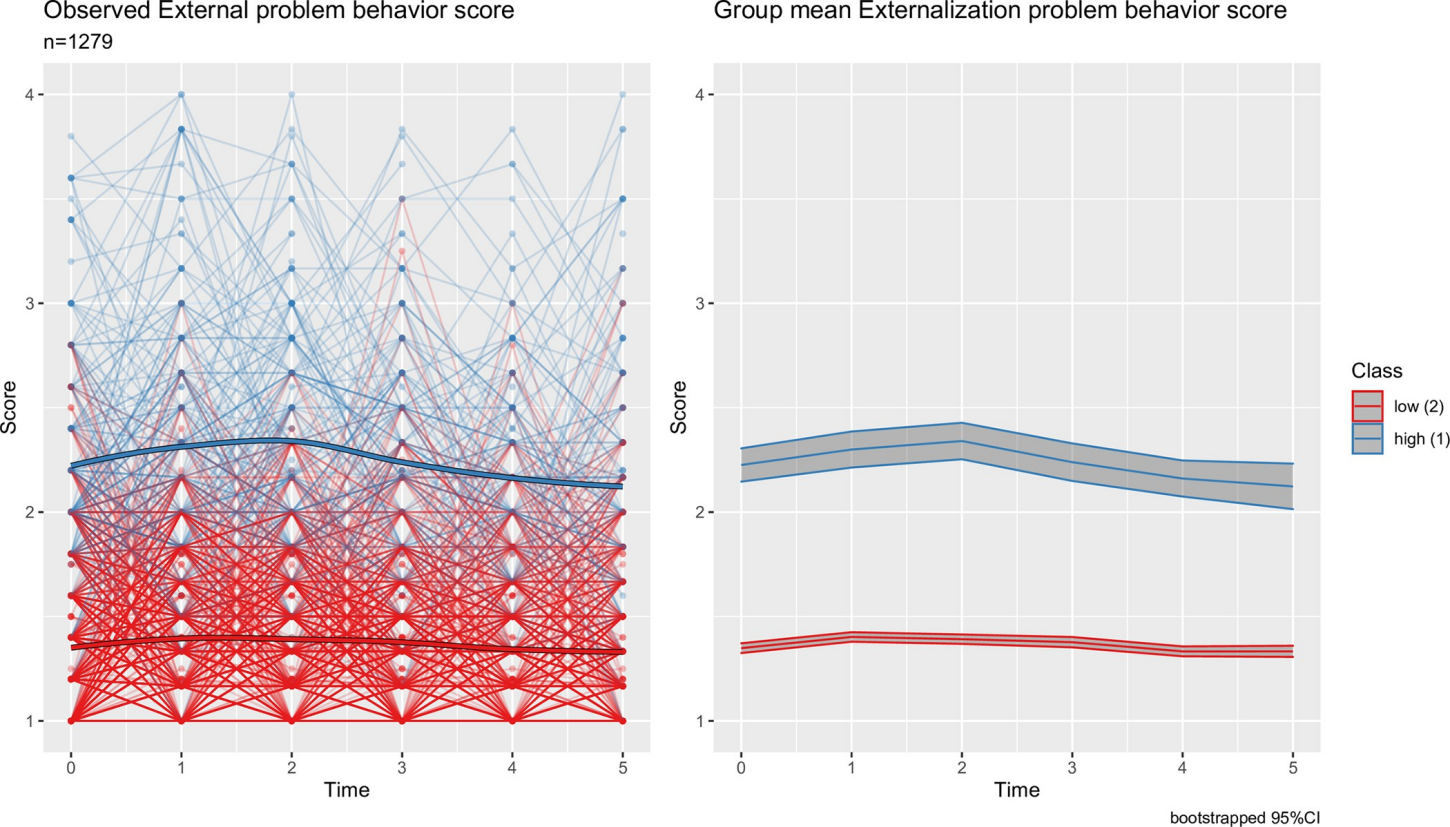
Longitudinal trajectories of externalizing problem behavior scoresLeft panel shows individual observed trajectories with dark line representing the mean for each latent class. Right panel shows class mean with shaded region representing the bootstrapped 95% confidence band.

**Fig 2:**
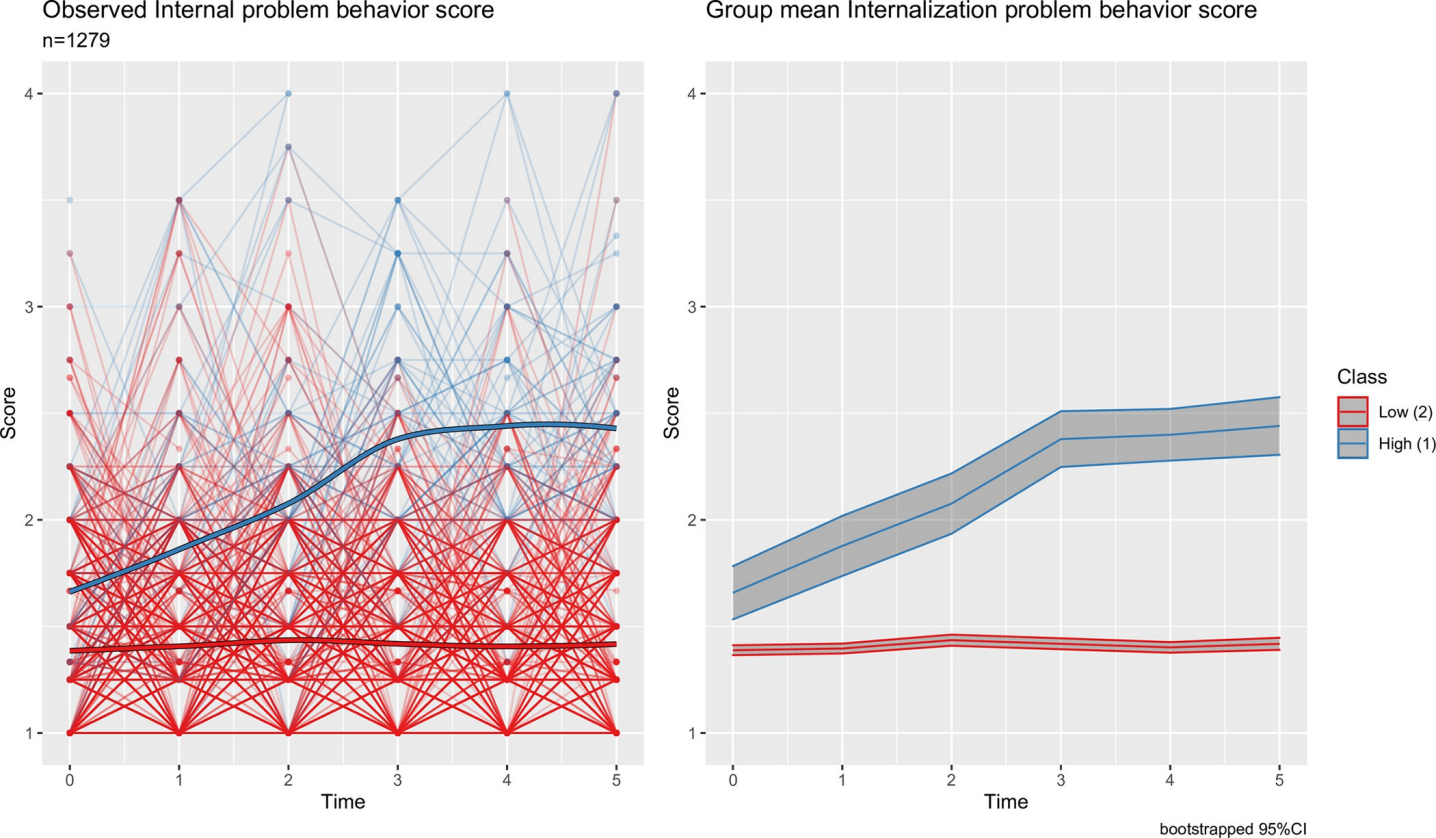
Longitudinal trajectories of internalizing problem behavior scores. Left panel shows individual observed trajectories with dark line representing the mean for each latent class. Right panel shows class mean with shaded region representing the bootstrapped 95% confidence band.

Figs 1 and 2 show trajectories of the scores for externalizing and internalizing problem behaviors with latent classes selected from the best fitting models. For both externalizing and internalizing problem behavior trajectories, a two-class solution was optimal. For each problem behavior trajectory, the latent classes identified from the LCMMs were labelled as high- and low-risk groups based on the relative group means of the observed scores among children within each class. The high-risk group for externalizing problem behavior consisted of 16.3%(n = 208) of the sample, with the average score of 2.24 (SD = 0.57). Whereas, the low-risk group had 83.7%(n = 1071) of the children with the overall average score of 1.37 (SD = 0.36). Further, the trajectories of scores for both high- and low-risk groups for externalizing problem were stationary over time. Class membership for internalizing problem behavior was more imbalanced across groups, with 6.9%(n = 88) of children in the high-risk group and 93.1%(n = 1191) of children in the low-risk group. In contrast to the externalizing problem behavior, the trajectory of the high-risk group for internalizing problem behavior displayed an increasing trend over time, with the average scores of 1.66 (SD = 0.55) in kindergarten and 2.44 (SD = 0.55) in fifth grade, respectively. Whereas, the low-risk group for the internalizing problem behavior displayed a stationary trajectory with an overall average score of 1.41 (SD = 0.39).

Sample characteristics of the children in high- and low-risk classes for each of the problem behaviors are also reported in S1 Table. Descriptively, among those in high-risk subgroups for both problem behaviors, there were lower percent of females and higher percent of children with disability. Further, compared to the low-risk subgroups, high-risk subgroups reported higher math, reading and science scores, and scored lower on teacher rated sub-scales for interpersonal skills, self-control, inhibitory control and attention focus.

### Prediction of children at high-risk of problem behaviors

**Fig 3:**
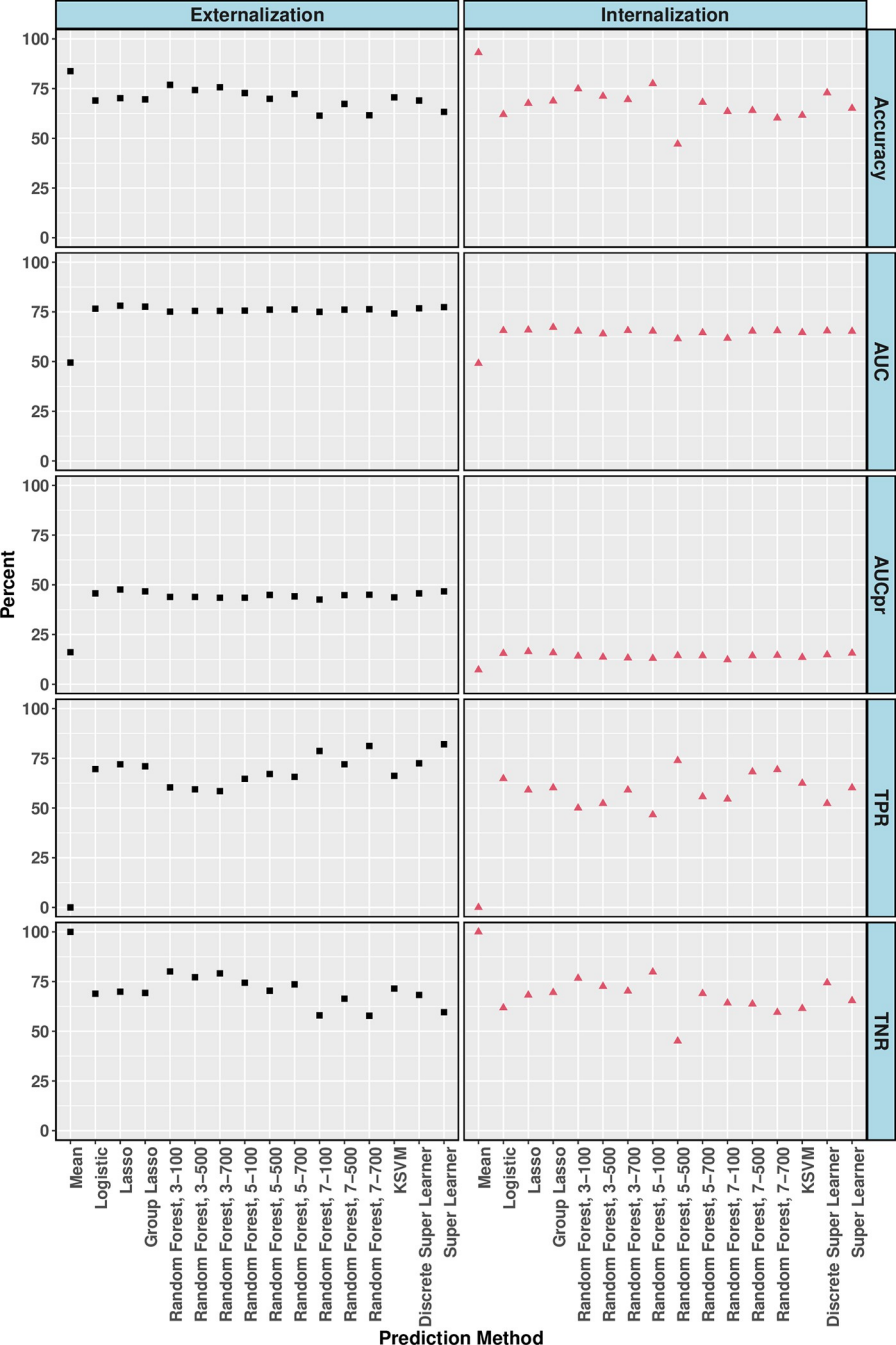
Crossvalidated performance metrics at optimal threshold for predicting externalizing problem behavior (externalization; left panel) and internalizing problem behavior (internalization; right panel); using predictions from Superlearner ensemble as well as individual candidate algorithms. Individual candidate algorithms included mean learner (Mean), logistic regression (Logistic), lasso regression (Lasso), group lasso regression (Group Lasso,) random forest with different combinations of number of variables sampled at each split and number of trees, and support vector machine with radial basis kernel function (KSVM). ACU = area under the curve, AUCpr = area under the precision recall curve, TPR = true positive rate, TNR = true negative rate.

Comparison of the performance metrics for the predictions from the Superlearner and individual candidate algorithms is shown in Fig 3. For the externalizing problem behavior outcome, compared to the mean learner, all machine learning candidate algorithms had better predictive performance. While the AUCpr of mean learner was lower than the baseline (AUCpr = 0.161 vs 0.163), AUCpr for all other algorithms were relatively higher ranging from 0.42 to 0.48, and lasso was the best algorithm overall (AUCpr = 0.48) followed by SuperLearner (AUCpr = 0.47). The AUC, ranging between 0.75 and 0.78, ranked algorithms similarly, with lasso performing best (AUC = 0.78) followed by Superlearner (AUC = 0.77). Lasso regression also provided the best prediction in terms of balancing TPR and TNR (72% and 69%) with accuracy of 70% at optimal cutoff of 0.154. Superlearner had an accuracy of 63%, TPR of 82% and TNR of 60% at optimal cutoff of 0.123. Further, performance of logistic regression was comparable in terms of AUC (0.77), AUCpr (0.46) and TNR (69%), with slightly lower accuracy (69%) and TPR (69%).

In predicting internalizing problem behavior, where the target high-risk class was rare, there was disagreement between AUCpr and AUC in identifying the best algorithm. All algorithms had an AUCpr higher than the baseline value of 0.069, ranging between 0.12 and 0.16, with the mean learner performing the worst. Lasso had the best AUCpr (0.164), followed by Superlearner (AUCpr = 0.158). Overall, AUC ranged between 0.62 (for logistic regression) and 0.67 (for Superlearner). Superlearner also had the overall best balance of TPR (60%) and TNR (69%) with an accuracy of 69% at the optimal cutoff of 0.072. Compared to other algorithms, logistic regression had lower AUC (0.62), AUCpr (0.12), accuracy (64%) and TPR (55%).

For predicting externalizing problem behavior, all algorithms except the mean learner and logistic regression had a good calibration (S1 Fig), with the best calibration for the ensemble Superlearner, indicating that the predicted risk scores were representative of the observed risk categories. However, most of the learners in the prediction of internalizing problem behavior were not well-calibrated, given that the outcome was rare. Superlearner ensemble was also comparably better calibrated for predicting internalizing problem behavior.

**Fig 4:**
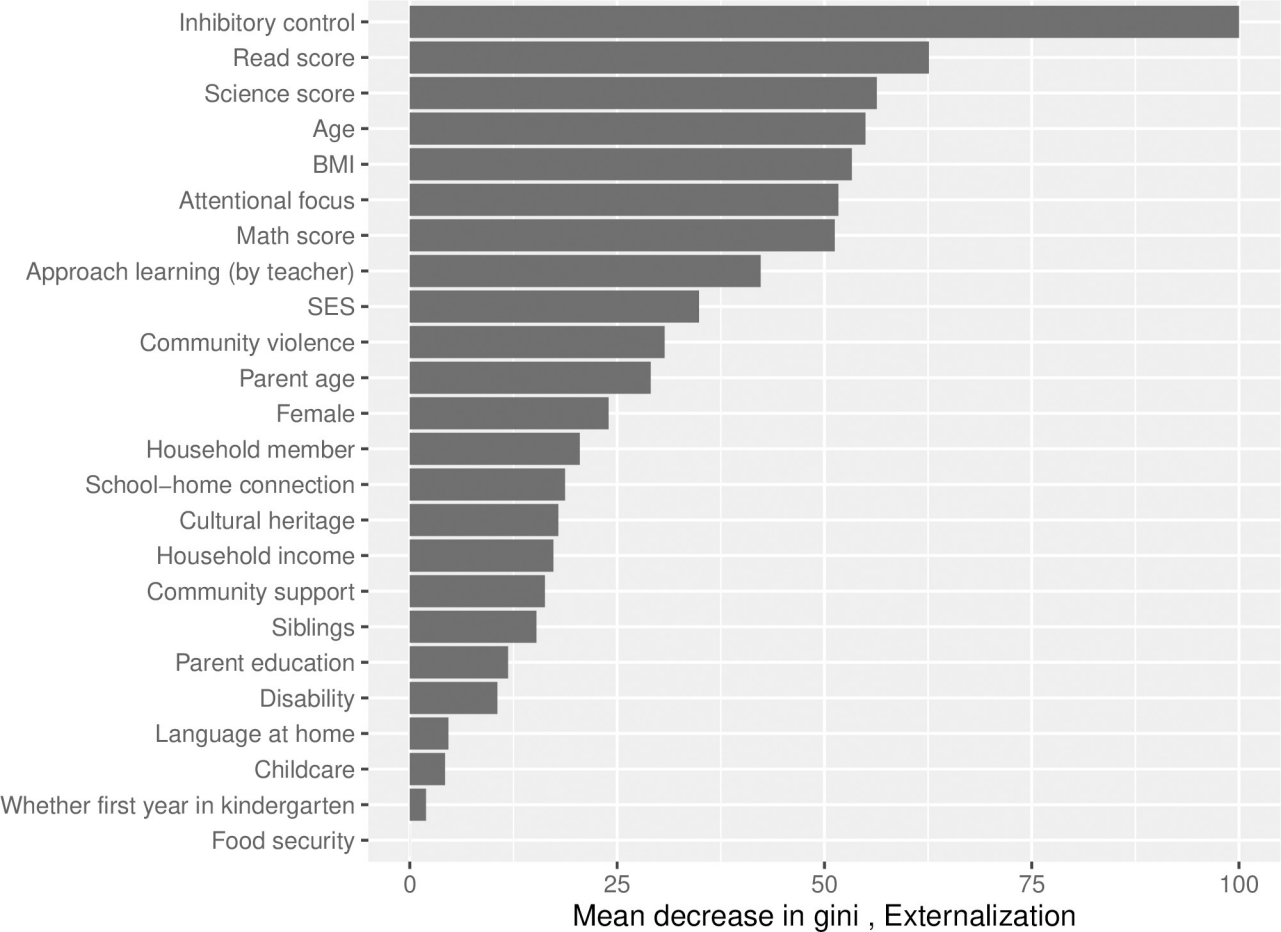
Variable importance metric as measured by mean decrease in gini index (standardized between 0 and 100) for externalizing problem behavior, based on random forest with the number of variables sampled at each split (mtry) = 7 and number of trees (ntree) = 700 fitted on the entire dataset. BMI = body mass index, SES = socioeconomic status.

**Fig 5:**
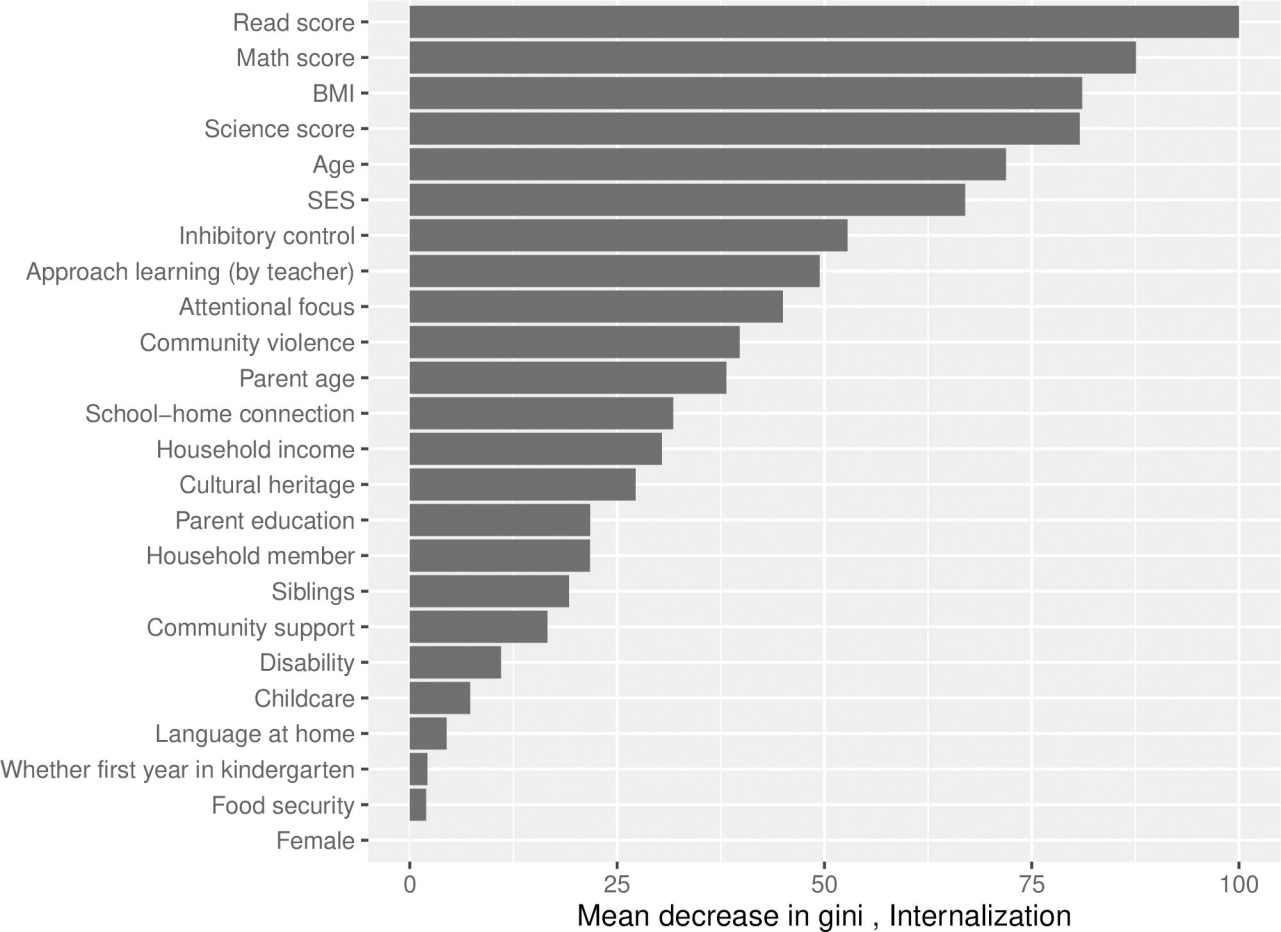
Variable importance metric as measured by mean decrease in gini index (standardized between 0 and 100) for internalizing problem behavior, based on random forest with the number of variables sampled at each split (mtry) = 5, and number of trees (ntree) = 100 fitted on the entire dataset. BMI = body mass index, SES = socioeconomic status.

Finally, for computing variable importance for externalizing problem behavior outcome, among different parameter settings for the random forest algorithm, mtry of 7 and ntree of 700 with the optimized AUC of 0.763 were selected. The top ten important baseline predictors based on variable importance measures (Fig 4) were combination of teacher-rated scores (inhibitory control, attentional focus and approach to learning), test scores (science score, reading score, math score), child’s age, BMI and social determinants including SES and community violence. For internalizing problem behavior outcome, the random forest algorithm with mtry of 5 and ntree 100 had optimized AUC of 0.656, and was selected for computing variable importance (Fig 5). Similar to the externalizing problem behavior, the top ten important early childhood predictors for the internalizing problem behavior were test scores (reading, math and science), child-specific factors (age and BMI), teacher-rated scores on inhibitory control, approach to learning, attentional focus and social determinants (SES and community violence).

**Fig 6:**
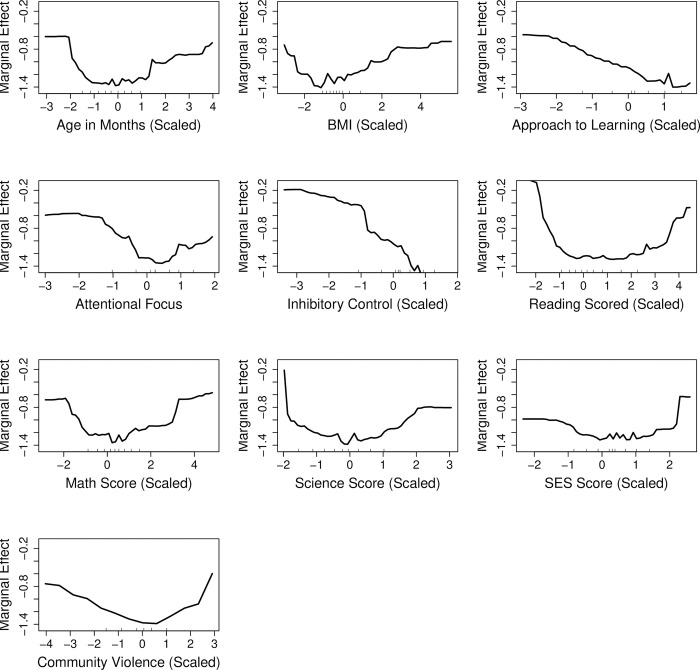
Partial dependence plots showing the dependence of the top ten important variables with prediction of externalizing problem behaviors. The y-axis represents the log odds of predicted probability for a fixed value of the variable of interest, conditional on all other variables (marginal effect). The x-axis represents the observed values of the variables scaled to have mean of zero and standard deviation of 1. BMI = body mass index, SES = socioeconomic status.

**Fig 7:**
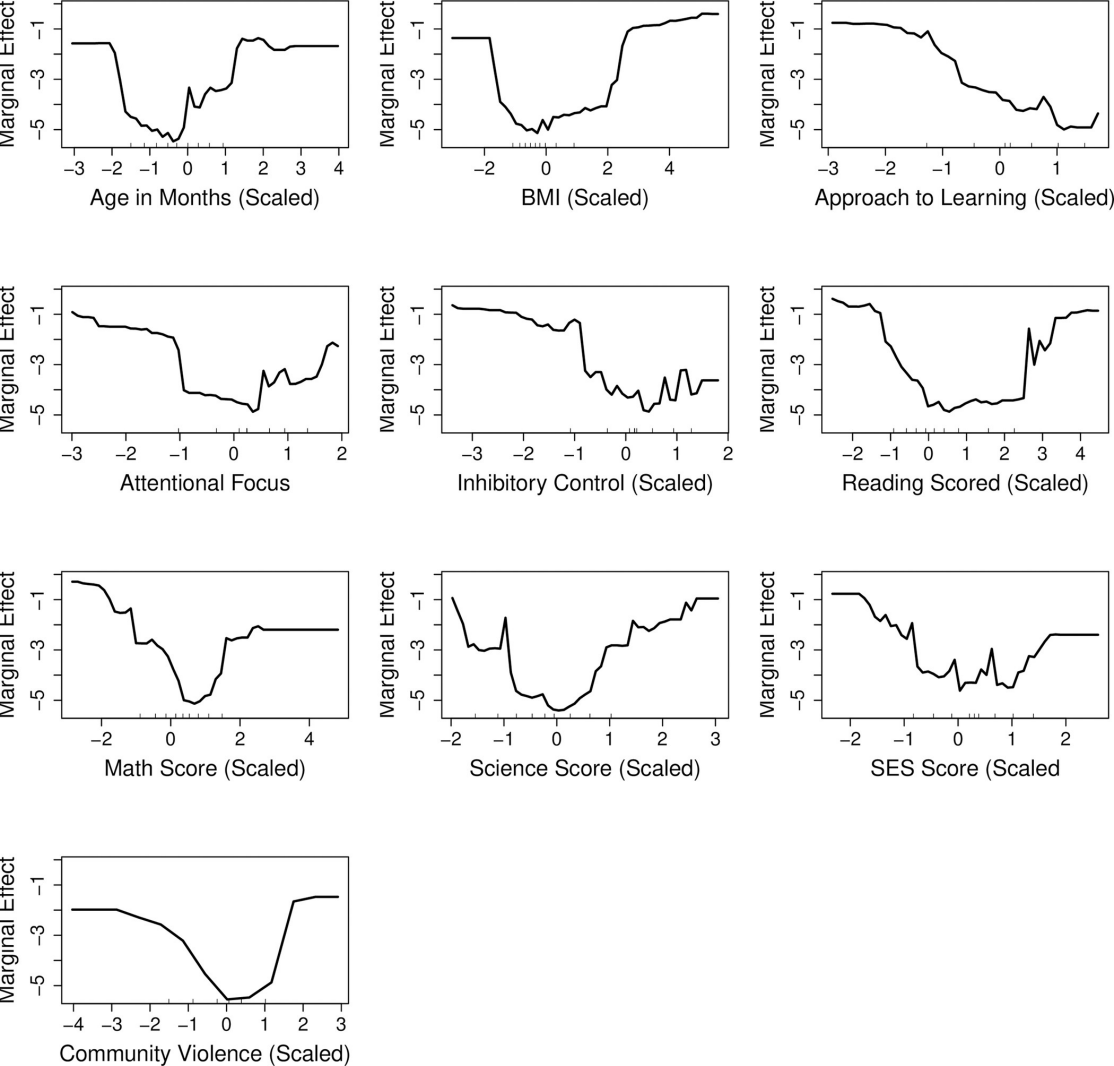
Partial dependence plots showing the dependence of the top ten important variables with prediction of internalizing problem behaviors. The y-axis represents the log odds of predicted probability for a fixed value of the variable of interest, conditional on all other variables (marginal effect). The x-axis represents the observed values of the variables scaled to have mean of zero and standard deviation of 1. BMI = body mass index, SES = socioeconomic status.

Figs 6 and 7 show the partial dependence plots for the top ten variables identified by variable importance measure for externalizing and internalizing problem behaviors, respectively. For externalizing problem behavior, the patterns of associations with predicted probability of high-risk were similar for age and BMI. Age (in months) and BMI showed non-linear associations such that the values on the low and high end of the distribution of the variables had higher predicted risks than those in the center. Higher scores for approach to learning, attentional focus and inhibitory control were contributing to lower predicted risk. Finally, scores on reading math and science, as well as SES and community violence showed inverted-U associations with predicted risks. The shapes of the associations were similar for predicting increased risk of high internalizing problem behavior.

## Discussion

We explored several state-of-the-art data-driven analytical methods and assessed the value of using machine learning for health disparities research. As a case example, we focused on Asian American children’s mental health problems. We used big data from the US ECLS-K 2010–2011 longitudinal cohort and applied machine learning to study two specific research questions. We first utilized unsupervised machine learning to identify clusters of mental health developmental trajectories for childhood internalizing and externalizing problem behaviors. Next, we utilized supervised machine learning to identify children at high-risk of problem behavior development and related top-ranking predictors from early childhood.

Among the Asian American cohort considered in our study, the high-risk group we identified for externalizing problem behavior had a constant trajectory with the mean score of 2.24. Whereas, the high-risk group for internalizing problem behavior had a mean score of 1.66 in kindergarten which increased to 2.44 in grade five. However, the overall average in the entire ECLS-K sample, as reported by NCES [29], ranged between 1.61 and 1.78 for externalizing problem behavior, and between 1.47 and 1.69 for internalizing problem behavior, from kindergarten to grade five. Thus, even though clinical cutoffs for the externalizing and internalizing problem behaviors measures have not been established, the high-risk subgroups we identified using data-driven approach expressed on average higher mental health burden compared to the reported averages for the overall ECLSK-2011 sample. This finding is especially significant because poor mental health, including internalizing and externalizing problem behaviors, is associated with various behavioral problems among youth, including lower educational achievements and increased engagement in risky behaviors, and these problems often persist into adulthood [30–32].

Consistent with the literature, we found 10–20% of Asian American children were at high risk for externalizing problems [33], and the externalizing problems persisted or slightly reduced over time [34, 35]. Similarly, consistent with the internalizing behavior literature based on teacher-reported data, compared to externalizing problems we found lower proportion of children (less than 10%) were at risk for internalizing problem. Literature has shown that the proportion of Asian American children with internalizing problems is often higher from parent-reported data (>20%), due to challenges in observing less expressive behaviors and symptoms by teachers [36]. We also observed that the internalizing problem emerged over time as children became more mature in cognitive and self-identity development with age [34, 37]. The findings from the internalizing problem behavior trajectory analysis suggest that prior to 3^rd^ grade (around age 8) is the critical period for early intervention to prevent deterioration of internalizing problems in Asian American children. Further, similar analyses incorporating parent reported outcomes are needed to address existing mental health need of Asian American children.

In the prediction analysis, we compared several machine learning algorithms with the traditional logistic regression. We included child, parent, family, and social determinants at kindergarten as predictors. As our primary algorithm, we considered a flexible ensemble approach (Superlearner), which was constructed using predictions from multiple algorithms ranging from traditional logistic regression to more complex ones such as random forest and support vector machine. We evaluated individual algorithms as well as the Superlearner. Consistent with the literature [38], we demonstrated that data-driven machine learning algorithms such as Superlearner and lasso regression have the potential to predict mental health outcomes with acceptable to high AUC (>0.75 for externalizing and >0.67 for internalizing problem behaviors). Further, predictions from the Superlearner had the best calibration for both mental health outcomes, suggesting that predicted probabilities were more representative of the observed risk patterns. Our results demonstrate the feasibility and usefulness of using data-driven machine learning to identify and uncover predictors without pre-specifying functional form or transformations of covariates, when complex multilevel predictors are considered and when there are limited theoretical frameworks to guide decision making. However, these models are not yet suitable for clinical utilization given the sub-optimal accuracy and true positive rates, as well as limited external validity.

Based on the variable importance measures, we identified similar predictors for externalizing and internalizing problem behaviors. The most important predictors for both outcomes were school readiness/early learning competency (reading, math and science scores at kindergarten), child-specific factors (age and BMI), teacher-rated scores on inhibitory control, approach to learning, attentional focus, and social determinants of health (SES and community violence). Our findings are mostly consistent with the developmental literature that school readiness (including emotion regulation/control, early learning and foundational academic skills) and living contexts (i.e., poverty and community violence) are critical predictors for childhood problem behavior development [6, 12, 37]. We found Asian American children’s early school readiness, learning behavior, emotion and behavioral regulations are key contributing factors for growing or persistence of both internalizing and externalizing problems. We also found SES, community violence and cultural heritage are important for problem behaviors development. These findings further confirm the benefit of early intervention programs that target on school readiness, family SES needs, cultural identity, and neighborhood/community violence. However, somewhat unexpectedly, we found child BMI as an important predictor that is worth further investigation to better understand underlying mechanisms. Association between BMI and perceived weight with mental health among adolescents have been established, showing that lower and higher than normal BMI are associated with high mental health risk [39]. However, previous research has not focused on health differences in mental health problem development among young Asian American children [40, 41]. Our findings inform new direction of mental health disparities research to better understand related experience in Asian American children.

This study contributes to mental health disparities research in several innovative ways. From the analytical perspective, our study demonstrates the value of applying the LCMM to uncover underlying patterns of trajectories, and supervised machine learning algorithms to identify predictors that have not been discovered in previous Asian American mental health research. From the health disparities evidence perspective, this study also contributes new evidence to guide future directions of psychopathology and intervention research for improving Asian American youths’ mental health. Specifically, our analysis uncovered a subgroup of Asian American children at an elevated risk of mental health disorders, as well as informed the critical age period for early intervention. The prediction modeling helped to identify children at risk and the important factors that need to be prioritized for early intervention.

Although this study demonstrates the feasibility of machine learning for health disparities research, there are few limitations that need to be considered. First, while machine learning methods are more flexible, can accommodate high number of covariates and require fewer model assumptions in terms of linearity and interactions, they often come with a cost of reduced interpretability. In our case study, the gain in performance from machine learning was only marginal compared to logistic regression. However, with a high number of correlated covariates, logistic regression often runs into stability issues and may not provide consistent results, which is not the case with machine learning algorithms. While we attempted to open the black box by looking at the variable importance metrics and partial dependence plots, we need to be careful not to make any causal interpretation from such results. Further, partial dependence plots are not suitable to visualize interaction and joint associations between multiple covariates and outcome of interest, and thus may not always reflect the full picture of dependence. Second, this study included a limited set of previously identified factors as predictors. For the exploration purpose, we did not include many parenting, school/classroom, and cultural/racial contextual factors that have also been identified in childhood mental health literature.

It is important to consider both traditional theory-guided epidemiological research and new machine learning methods to advance health disparities research. It is necessary to build on the current work and consider other parenting, family, cultural/racial contextual factors, time variant, developmental stages/ages, and dynamic influence of contextual factors on mental health trajectory development among Asian American children. While our study was focused on assessing longitudinal trajectory and predicting risk of persistent symptomatology over time to inform early intervention strategies, an alternative approach assessing high-risk children at kindergarten may provide additional insights. However, children who demonstrate persistent or increasing trajectory of high problem behaviors during their developmental years, beyond kindergarten, will benefit tremendously from early mental healthcare intervention. Thus, our approach provides a foundation for addressing current mental health crisis by first assessing trends of problem behaviors over the developmental years rather than at a static time point. Further, to better inform whether similar analytical approach developed in this study and findings can be replicated to other racial/ethnic groups, our long-term goal is to apply a similar methodology to other populations and develop a generalizable analytic framework for future mental health disparity research among different racial/ethnic groups.

This study demonstrates the feasibility and value of using machine learning for mental health research. We uncover new patterns and predictors that are important to Asian American children’s mental health. Several clinical implications can be drawn from our study. Findings from the cluster trajectory analysis suggest that critical age for early intervention for externalizing problem needs to be prior to kindergarten (before time 0 when the group difference is not established), and for internalizing problem needs to be between kindergarten and age 8 (time 0–4, during the time group difference starting to emerge). Findings from the prediction analysis also suggest potential benefit of applying evidence-based interventions that aim to promote early school readiness, culture identity, and reduce adversity of family SES and neighborhood/ community violence to improve mental health outcomes for Asian American children. Finally, our study suggests the need for additional epidemiological mechanism and causal pathway testing research for the predictors that have not been well studied to inform new strategies to improve mental health outcomes among Asian American children.

## Supporting information

S1 Table Summary of baseline characteristics of Asian American children in the longitudinal cohort, overall and by latent cluster classes for internalizing problem behavior and externalizing problem behavior. (DOCX)

S2 Table Summary of missing observations for continuous measures considered as candidate predictors. (DOCX)

S1 Fig Calibration plots comparing observed and predicted risk scores within each decile of predicted risk interval for externalizing problem behavior outcome. (PDF)

S2 Fig Calibration plots comparing observed and predicted risk scores within each decile of predicted risk interval for internalizing problem behavior outcome. (PDF)
