# Supplementary material for: Applied machine learning to identify differential risk groups underlying externalizing and internalizing problem behaviors trajectories: A case study using a cohort of Asian American children
Source: PLoS One. 2023 Mar 3;18(3):e0282235. doi: 10.1371/journal.pone.0282235 (PMC9983857; doi:10.1371/journal.pone.0282235)
Supplement: S1 Table — (DOCX) [file pone.0282235.s001.docx]

| **S1 Table: Summary of baseline characteristics of Asian American Children in the longitudinal cohort, overall and by latent cluster classes for internalizing problem behavior and externalizing problem behavior.** | | | | | |
| --- | --- | --- | --- | --- | --- |
|  |  |  |  |  |  |
|  | **Overall (N = 1279)** | **Internalizing problem behavior** | | **Externalizing problem behavior** | |
|  |  | **Low (N = 1191)** | **High (N = 88)** | **low (N = 1071)** | **high (N = 208)** |
| **Child’s Age, mean (sd) months** | 72.36 (4.23) | 72.29 (4.23) | 73.25 (4.13) | 72.39 (4.21) | 72.20 (4.32) |
| **Gender, n (%)** |  |  |  |  |  |
| **Female** | 679 (53.1%) | 639 (53.7%) | 40 (45.5%) | 627 (58.5%) | 52 (25.0%) |
| **Male** | 600 (46.9%) | 550 (46.2%) | 48 (54.5%) | 443 (41.4%) | 155 (74.5%) |
| **Missing** | 2 (0.2%) | 2 (0.2%) | 0 | 1 (0.1%) | 1 (0.5%) |
| **BMI (scaled), mean (sd)** | -0.18 (0.96) | -0.19 (0.94) | -0.01 (1.28) | -0.19 (0.97) | -0.12 (0.93) |
| **Disability, n(%)** |  |  |  |  |  |
| Yes | 89 (7.0%) | 75 (6.3%) | 14 (15.9%) | 57 (5.3%) | 32 (15.4%) |
| No | 831 (65.0%) | 785 (65.9%) | 46 (52.3%) | 727 (67.9%) | 104 (50.0%) |
| Missing | 359 (28.1%) | 331 (27.8%) | 28 (31.8%) | 287 (26.8%) | 72 (34.6%) |
| **1st year in kindergarten, n(%)** |  |  |  |  |  |
| No | 48 (3.8%) | 45 (3.8%) | 3 (3.4%) | 38 (3.5%) | 10 (4.8%) |
| Yes | 963 (75.3%) | 898 (75.4%) | 65 (73.9%) | 809 (75.5%) | 154 (74.0%) |
| Missing | 268 (21.0%) | 248 (20.8%) | 20 (22.7%) | 224 (20.9%) | 44 (21.2%) |
| **Reading score (scaled), mean(sd)** | 0.41 (1.25) | 0.44 (1.23) | 0.01 (1.36) | 0.47 (1.23) | 0.10 (1.29) |
| **Math score (scaled), mean(sd)** | 0.35 (1.00) | 0.38 (0.99) | -0.08 (1.03) | 0.38 (0.99) | 0.19 (1.04) |
| **Science score (scaled), mean(sd)** | -0.22 (0.98) | -0.20 (0.97) | -0.46 (1.08) | -0.19 (0.97) | -0.36 (1.03) |
| **Language spoken at home, n(%)** |  |  |  |  |  |
| Non-English | 613 (47.9%) | 572 (48.0%) | 41 (46.6%) | 518 (48.4%) | 95 (45.7%) |
| English | 481 (37.6%) | 446 (37.4%) | 35 (39.8%) | 411 (38.4%) | 70 (33.7%) |
| Missing | 185 (14.5%) | 173 (14.5%) | 12 (13.6%) | 142 (13.3%) | 43 (20.7%) |
| **Parent's education, n(%)** |  |  |  |  |  |
| Less than high school | 69 (5.4%) | 64 (5.4%) | 5 (5.7%) | 56 (5.2%) | 13 (6.2%) |
| Some high school | 345 (27.0%) | 320 (26.9%) | 25 (28.4%) | 286 (26.7%) | 59 (28.4%) |
| College | 340 (26.6%) | 313 (26.3%) | 27 (30.7%) | 288 (26.9%) | 52 (25.0%) |
| Graduate | 338 (26.4%) | 319 (26.8%) | 19 (21.6%) | 295 (27.5%) | 43 (20.7%) |
| Missing | 187 (14.6%) | 175 (14.7%) | 12 (13.6%) | 146 (13.6%) | 41 (19.7%) |
| **Household income, n(%)** |  |  |  |  |  |
| 0-20K | 138 (10.8%) | 125 (10.5%) | 13 (14.8%) | 116 (10.8%) | 22 (10.6%) |
| 20K-45K | 219 (17.1%) | 200 (16.8%) | 19 (21.6%) | 185 (17.3%) | 34 (16.3%) |
| 45K, 100K | 303 (23.7%) | 284 (23.8%) | 19 (21.6%) | 259 (24.2%) | 44 (21.2%) |
| 100K,200K | 235 (18.4% | 228 (19.1%) | 7 (8.0%) | 198 (18.5%) | 37 (17.8%) |
| 200K+ | 73 (5.7%) | 66 (5.5%) | 7 (8.0%) | 63 (5.9%) | 10 (4.8%) |
| Missing | 311 (24.3%) | 288 (24.2%) | 23 (26.1%) | 250 (23.3%) | 61 (29.3%) |
| **SES, scaled mean (sd)** | 0.29 (0.81) | 0.30 (0.81) | 0.19 (0.77) | 0.31 (0.82) | 0.21 (0.79) |
| **Food security, n(%)** |  |  |  |  |  |
| Secure | 849 (66.4%) | 796 (66.8%) | 53 (60.2%) | 723 (67.5%) | 126 (60.6%) |
| Low | 61 (4.8%) | 54 (4.5%) | 7 (8.0%) | 52 (4.9%) | 9 (4.3%) |
| Missing | 369 (28.9%) | 341 (28.6%) | 28 (31.8%) | 296 (27.6%) | 73 (35.1%) |
| **HH members, n(%)** |  |  |  |  |  |
| 2 | 12 (0.9%) | 12 (1.0%) | 0 | 12 (1.1%) | 0 |
| 3 | 124 (9.7%) | 114 (9.6%) | 10 (11.4%) | 92 (8.6%) | 32 (15.4%) |
| 4 | 416 (32.5%) | 385 (32.3%) | 31 (35.2%) | 357 (33.3%) | 59 (28.4%) |
| 5 | 213 (16.7%) | 199 (16.7%) | 14 (15.9%) | 182 (17.0%) | 31 (14.9%) |
| 6 | 115 (9.0%) | 108 (9.1%) | 7 (8.0%) | 104 (9.7%) | 11 (5.3%) |
| 7+ | 88 (6.9%) | 85 (7.1%) | 3 (3.4%) | 74 (6.9%) | 14 (6.7%) |
| Missing | 311 (24.3%) | 288 (24.2%) | 23 (26.1%) | 250 (23.3%) | 61 (29.3%) |
| **Number of Siblings, n(%)** |  |  |  |  |  |
| 0 | 170 (13.3%) | 159 (13.4%) | 11 (12.5%) | 132 (12.3%) | 38 (18.3%) |
| 1 | 515 (40.3%) | 473 (39.7%) | 42 (47.7%) | 448 (41.8%) | 67 (32.2%) |
| 2 | 204 (15.9%) | 197 (16.5%) | 7 (8.0%) | 177 (16.5%) | 27 (13.0%) |
| 3 | 53 (4.1%) | 50 (4.2%) | 3 (3.4%) | 42 (3.9%) | 11 (5.3%) |
| 4+ | 26 (2.0%) | 24 (2.0%) | 2 (2.3%) | 22 (2.1%) | 4 (1.9%) |
| Missing | 311 (24.3%) | 288 (24.2%) | 23 (26.1%) | 250 (23.3%) | 61 (29.3%) |
| **Childcare, n(%)** |  |  |  |  |  |
| Other | 595 (46.5%) | 561 (47.1%) | 34 (38.6%) | 500 (46.7%) | 95 (45.7%) |
| Relative | 159 (12.4%) | 145 (12.2%) | 14 (15.9%) | 138 (12.9%) | 21 (10.1%) |
| Missing | 525 (41.0%) | 485 (40.7%) | 40 (45.5%) | 433 (40.4%) | 92 (44.2%) |
| **Home-child interaction, mean (sd)** | 2.85 (0.48) | 2.85 (0.49) | 2.92 (0.38) | 2.86 (0.48) | 2.84 (0.52) |
| **Reading together, mean (sd)** | 3.26 (0.57) | 3.25 (0.58) | 3.35 (0.47) | 3.27 (0.57) | 3.19 (0.61) |
| **Home-school connection, mean (sd)** | 1.47 (0.36) | 1.47 (0.36) | 1.51 (0.40) | 1.46 (0.36) | 1.52 (0.35) |
| **Community violence, mean (sd)** | 3.86 (0.38) | 3.86 (0.38) | 3.88 (0.39) | 3.87 (0.37) | 3.82 (0.42) |
| **Cultural heritage, mean (sd)** | 3.32 (1.03) | 3.32 (1.03) | 3.25 (1.09) | 3.33 (1.04) | 3.26 (0.97) |
| **Approach to learn - parent, mean (sd)** | 3.09 (0.50) | 3.10 (0.50) | 2.95 (0.60) | 3.11 (0.50) | 2.95 (0.55) |
| **Approach to learn - teacher, mean (sd)** | 3.05 (0.57) | 3.08 (0.56) | 2.70 (0.64) | 3.13 (0.54) | 2.66 (0.59) |
| **Attentional focus, mean (sd)** | 5.00 (1.05) | 5.04 (1.03) | 4.48 (1.24) | 5.13 (0.98) | 4.34 (1.18) |
| **Social interaction - parent, mean (sd)** | 3.26 (0.65) | 3.27 (0.63) | 3.10 (0.79) | 3.27 (0.63) | 3.17 (0.74) |
| **Interpersonal - teacher, mean (sd)** | 2.99 (0.65) | 3.03 (0.63) | 2.48 (0.65) | 3.09 (0.61) | 2.52 (0.61) |
| **Self control - parent, mean (sd)** | 2.94 (0.47) | 2.94 (0.47) | 2.93 (0.45) | 2.96 (0.46) | 2.80 (0.50) |
| **Self control - teacher, mean (sd)** | 3.14 (0.62) | 3.17 (0.60) | 2.60 (0.66) | 3.25 (0.56) | 2.55 (0.57) |
| **Inhibitory control - teacher, mean (sd)** | 5.11 (1.05) | 5.15 (1.03) | 4.53 (1.22) | 5.28 (0.93) | 4.22 (1.21) |
| **Community support, mean (sd)** | 4.29 (0.78) | 4.28 (0.77) | 4.38 (0.79) | 4.29 (0.77) | 4.29 (0.81) |
| **Parent age (primary), mean (sd) years** | 36.26 (4.74) | 36.18 (4.73) | 37.34 (4.72) | 36.23 (4.68) | 36.44 (5.07) |
| **Health scale, mean (sd)** | 1.69 (0.81) | 1.69 (0.81) | 1.72 (0.77) | 1.67 (0.80) | 1.79 (0.82) |

BMI: body mass index

HH: household

SES: socio economic status score
