# Supplementary material for: Applied machine learning to identify differential risk groups underlying externalizing and internalizing problem behaviors trajectories: A case study using a cohort of Asian American children
Source: PLoS One. 2023 Mar 3;18(3):e0282235. doi: 10.1371/journal.pone.0282235 (PMC9983857; doi:10.1371/journal.pone.0282235)
Supplement: S2 Table — (DOCX) [file pone.0282235.s002.docx]

**S2 Table: Summary of missing observations for continuous measures considered as candidate predictors.**

| **Measures** | **Missing** | |
| --- | --- | --- |
|  | **N** | **%** |
| **Self-control - parent** | 532 | 41.60% |
| **Approach learning - parent** | 531 | 41.50% |
| **Social interaction** | 530 | 41.40% |
| **Child health** | 527 | 41.20% |
| **Home parent-child interaction** | 523 | 40.90% |
| **Reading together** | 523 | 40.90% |
| **Interpersonal** | 448 | 35.00% |
| **Self-control - teacher** | 447 | 34.90% |
| **Attentional focus** | 366 | 28.60% |
| **Inhibitory control** | 366 | 28.60% |
| **Approach learning - teacher** | 352 | 27.50% |
| **School-home connection** | 346 | 27.10% |
| **Cultural heritage** | 336 | 26.30% |
| **Parent’s age (primary)** | 328 | 25.60% |
| **SES** | 187 | 14.60% |
| **Community violence** | 181 | 14.20% |
| **Community support** | 140 | 10.90% |
| **Science score** | 75 | 5.90% |
| **Math score** | 67 | 5.20% |
| **BMI** | 47 | 3.70% |
| **Reading score** | 45 | 3.50% |
| **Child’s age** | 44 | 3.40% |
