# Supplementary material for: Applied machine learning to identify differential risk groups underlying externalizing and internalizing problem behaviors trajectories: A case study using a cohort of Asian American children
Source: PLoS One. 2023 Mar 3;18(3):e0282235. doi: 10.1371/journal.pone.0282235 (PMC9983857; doi:10.1371/journal.pone.0282235)
Supplement: S1 Fig — (PDF) [file pone.0282235.s003.pdf]

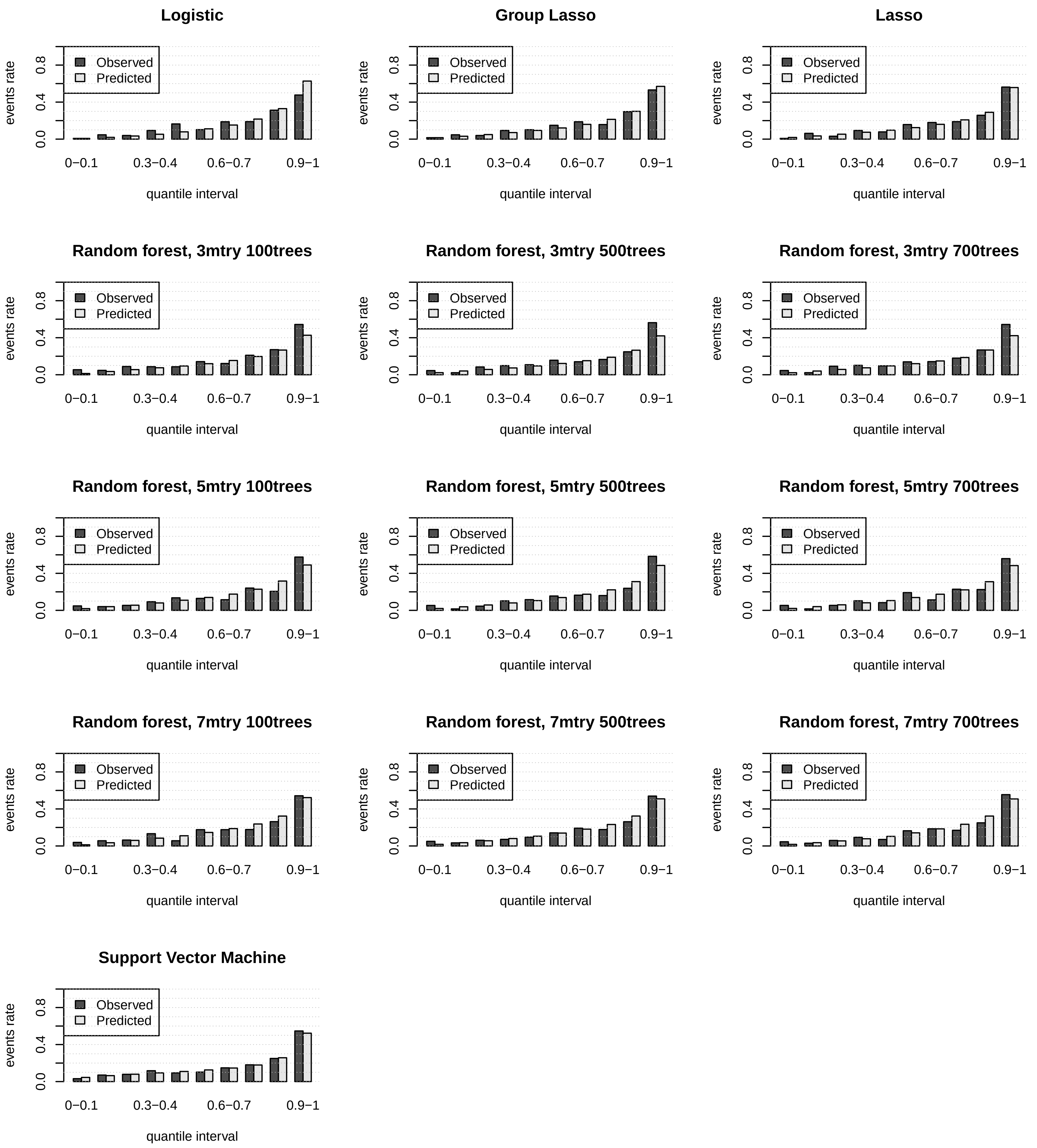
Logistic
Observed
Predicted
events rate
0.0
0.4
0.8
0−0.1
0.3−0.4
0.6−0.7
0.9−1
quantile interval
Group Lasso
Observed
Predicted
events rate
0.0
0.4
0.8
0−0.1
0.3−0.4
0.6−0.7
0.9−1
quantile interval
Lasso
Observed
Predicted
events rate
0.0
0.4
0.8
0−0.1
0.3−0.4
0.6−0.7
0.9−1
quantile interval
Random forest, 3mtry 100trees
Observed
Predicted
events rate
0.0
0.4
0.8
0−0.1
0.3−0.4
0.6−0.7
0.9−1
quantile interval
Random forest, 3mtry 500trees
Observed
Predicted
events rate
0.0
0.4
0.8
0−0.1
0.3−0.4
0.6−0.7
0.9−1
quantile interval
Random forest, 3mtry 700trees
Observed
Predicted
events rate
0.0
0.4
0.8
0−0.1
0.3−0.4
0.6−0.7
0.9−1
quantile interval
Random forest, 5mtry 100trees
Observed
Predicted
events rate
0.0
0.4
0.8
0−0.1
0.3−0.4
0.6−0.7
0.9−1
quantile interval
Random forest, 5mtry 500trees
Observed
Predicted
events rate
0.0
0.4
0.8
0−0.1
0.3−0.4
0.6−0.7
0.9−1
quantile interval
Random forest, 5mtry 700trees
Observed
Predicted
events rate
0.0
0.4
0.8
0−0.1
0.3−0.4
0.6−0.7
0.9−1
quantile interval
Random forest, 7mtry 100trees
Observed
Predicted
events rate
0.0
0.4
0.8
0−0.1
0.3−0.4
0.6−0.7
0.9−1
quantile interval
Random forest, 7mtry 500trees
Observed
Predicted
events rate
0.0
0.4
0.8
0−0.1
0.3−0.4
0.6−0.7
0.9−1
quantile interval
Random forest, 7mtry 700trees
Observed
Predicted
events rate
0.0
0.4
0.8
0−0.1
0.3−0.4
0.6−0.7
0.9−1
quantile interval
Support Vector Machine
Observed
Predicted
events rate
0.0
0.4
0.8
0−0.1
0.3−0.4
0.6−0.7
0.9−1
quantile interval
