# Supplementary material for: Applied machine learning to identify differential risk groups underlying externalizing and internalizing problem behaviors trajectories: A case study using a cohort of Asian American children
Source: PLoS One. 2023 Mar 3;18(3):e0282235. doi: 10.1371/journal.pone.0282235 (PMC9983857; doi:10.1371/journal.pone.0282235)
Supplement: S2 Fig — (PDF) [file pone.0282235.s004.pdf]

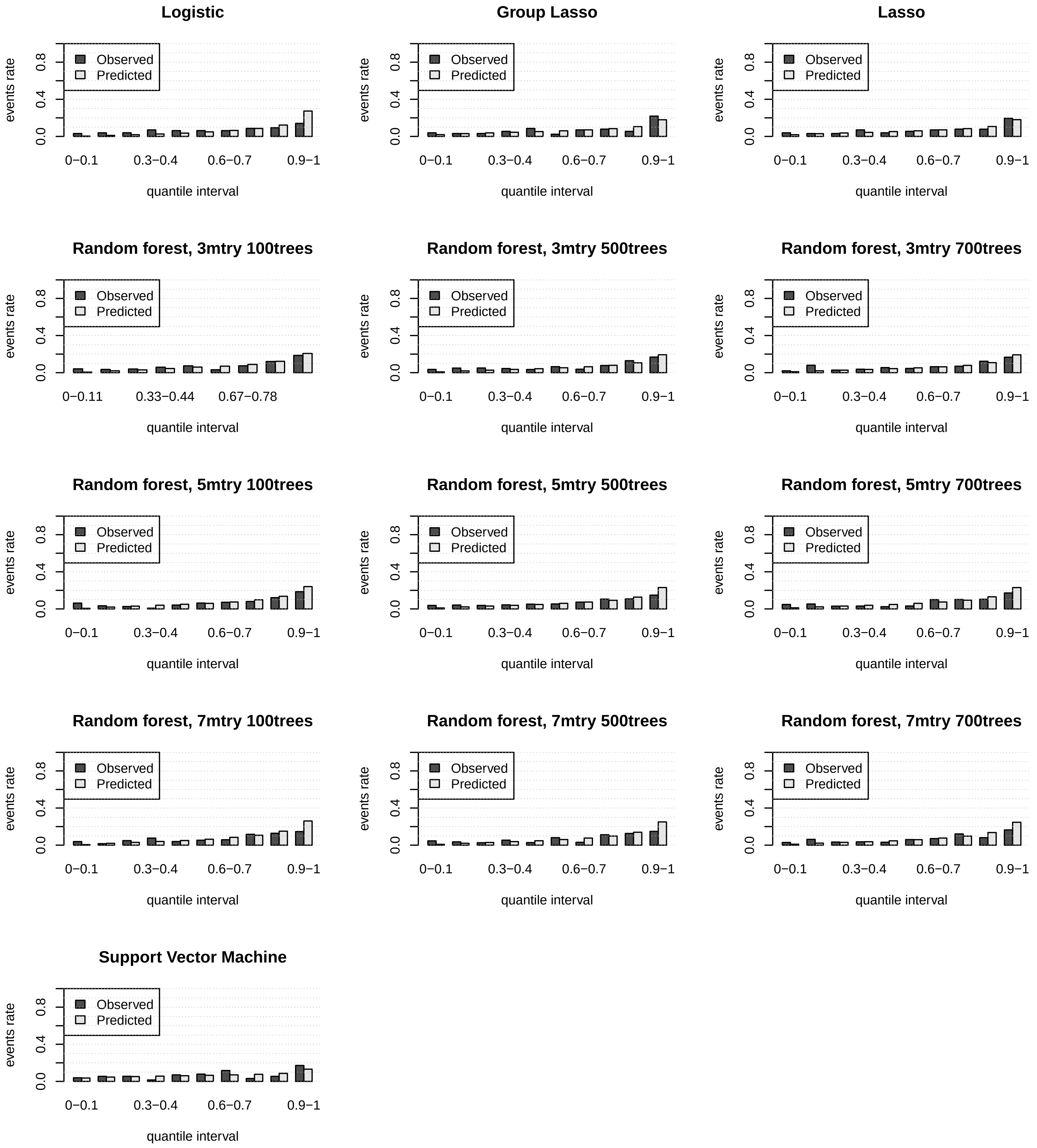
Logistic
Group Lasso
Lasso
Random forest, 3mtry 100trees
Random forest, 3mtry 500trees
Random forest, 3mtry 700trees
Random forest, 5mtry 100trees
Random forest, 5mtry 500trees
Random forest, 5mtry 700trees
Random forest, 7mtry 100trees
Random forest, 7mtry 500trees
Random forest, 7mtry 700trees
Support Vector Machine
Observed
Predicted
events rate
0.0
0.4
0.8
0−0.1
0.3−0.4
0.6−0.7
0.9−1
0−0.11
0.33−0.44
0.67−0.78
quantile interval
